# Influence of Type 1 Diabetes on the Symbolic Analysis and Complexity of Heart Rate Variability in Young Adults

**DOI:** 10.5935/abc.20180117

**Published:** 2018-07

**Authors:** Elaine Aparecida de Oliveira, Anne Kastelianne França da Silva, Diego Giuliano Destro Christofaro, Laís Manata Vanzella, Rayana Loch Gomes, Franciele Marques Vanderlei, Luiz Carlos Marques Vanderlei

**Affiliations:** Universidade Estadual Paulista Júlio de Mesquita Filho (UNESP), Presidente Prudente, SP - Brazil

**Keywords:** Diabetes Mellitus / complications, Diabetes / diagnosis, Diabetes / therapy, Young Adult, Heart Rate, Autonomic Nervous System

## Abstract

**Background:**

Type 1 diabetes mellitus can cause autonomic changes, which can be assessed by heart rate variability. Among the heart rate variability assessment methods, the symbolic analysis and Shannon entropy, based on the Chaotic dynamics, have gained prominence.

**Objective:**

To compare heart rate variability indexes, obtained through symbolic analysis and Shannon entropy, in young adults with type 1 diabetes mellitus and healthy young individuals, associated with the analysis of linear indexes; and to verify if there are associations between the indexes obtained by the symbolic analysis and by Shannon entropy and linear indexes in diabetic individuals.

**Methods:**

Heart rate variability data from 39 young adults with type 1 diabetes mellitus and 43 healthy young individuals were analyzed, using a cardio-frequency meter. Linear indexes (standard deviation of all normal RR intervals recorded in a time interval expressed in milliseconds; square root of the mean of the squared differences between adjacent normal RR intervals in a time interval expressed in milliseconds; low and high frequency components in millisecond squared; and normalized units and ratio between low and high frequency components) and nonlinear ones (Shannon entropy and symbolic analysis - standard without variation; with one or two variations; and with two different variations) of the heart rate variability were calculated. The statistical significance was set at 5%, and the confidence interval was 95%.

**Results:**

Significantly lower values were observed in the DM1 group compared to healthy young adults for the standard deviation indexes of all normal RR intervals recorded in a time interval [37.30 (29.90) vs. 64.50 (36.20); p = 0.0001], square root of the mean of the squared differences between adjacent normal RR intervals in a time interval [32.73 (17.43) vs. 55.59 (21.60); p = 0.0001], low frequency component [402.00 (531.00) vs. 1,203.00 (1,148.00); p = 0.0001], high frequency component [386.00 (583.00) vs. 963.00 (866.00); p = 0.0001] and the pattern with two different variations [15,33 (9,22) vs. 20.24 (12.73); p = 0.0114], with the effect of this difference being considered large (standard deviation of all normal RR intervals recorded in a time interval, square root of the mean of the squared differences between adjacent normal RR intervals in a time interval and low frequency component), medium (high frequency component) and small (standard with two different variations). The agreement of the associations between the linear and non-linear indexes was considered elevated for the high frequency component index - normalized units (r = -0.776), with the standard index without variation, and moderate for the indexes square root of the mean of the squared differences between adjacent normal RR intervals in a time interval (r = 0.550), standard deviation of all normal RR intervals recorded in a time interval (r = 0.522), high frequency component - normalized units (r = 0.638) with the index standard with two similar variations, as well as for the indexes square root of the mean of the squared differences between adjacent normal RR intervals in a time interval (r = 0.627) and high frequency component - normalized units (r = 0.601) with the index standard with two different variations.

**Conclusion:**

Type 1 diabetes mellitus influenced linear indexes and symbolic analysis, but not yet in the complexity of heart rate variability. Additionally, heart rate variability indexes correlated with the symbolic dynamics.

## Introduction

Type 1 diabetes mellitus (DM1) is characterized by the autoimmune destruction of insulin-producing cells, resulting from both environmental and genetic processes. This condition affects approximately 382 million people worldwide, affecting more frequently children, adolescents and young adults.^1^

Individuals with DM1 may have complications such as Cardiovascular Autonomic Neuropathy (CAN), caused by damage to autonomic nerve fibers associated with the cardiovascular system, resulting in neurohumoral regulation disorders.^2^ CAN may interfere with the individual’s quality of life and prognosis, showing some clinical manifestations such as resting tachycardia, exercise intolerance, stroke and sudden death of cardiac origin, among others.^3^

Heart Rate Variability (HRV), a simple, reproducible and non-invasive tool that shows the oscillations between consecutive heart beats (R-R intervals) associated with the Autonomic Nervous System (ANS) influence on the sinus node,^4^ is indicated for the early assessment of autonomic status in diabetic individuals.^5^ Its analyses can be performed by linear methods, in the time and frequency domains, and non-linear methods, in the chaotic domain,^6^ among which the symbolic analysis and entropy are highlighted.^7^

The symbolic analysis has been recently studied and stands out for being able to differentiate both components of ANS^6^ and quantify their impairment as a function of pathology, showing its effectiveness in assessing autonomic behavior and seeming appropriate to elucidate the neural pathophysiological mechanisms.^6,8^ Shannon Entropy (SE) demonstrates the degree of complexity of signal sample distribution,^9^ which allows identifying conditions that may interfere with cardiovascular regulation.^9^

Few studies have used these methods to assess individuals with diabetes.^10,11^ Using symbolic analysis to investigate individuals with type 2 diabetes mellitus (DM2) without CAN, Moura-Tonello et al.^10^ have indicated that this population has greater sympathetic modulation and reduced parasympathetic modulation and global variability. In subjects with DM2 and CAN, entropy analyses have shown the lower complexity of these individuals when compared to individuals with DM2 without CAN.^11^

HRV is a technique that allows the assessment of autonomic behavior and can be analyzed through linear and non-linear methods.^4^ It is also well established that HRV indexes may be altered in several conditions - among them diabetes mellitus. Furthermore, the literature indicates that SE and symbolic analysis, a new methodology for the analysis of HRV for autonomic behavior assessment, is altered in DM2, but the use of these HRV analysis methods in patients with DM1 is unknown.

Considering that DM1 more frequently affects children and young adults, and that this population is subject to several complications, including autonomic alterations, which may lead to CAN, studies such as this are necessary to identify whether the use of new methods of autonomic activity analysis are capable of observing changes in the ANS behavior of young adults with DM1 without CAN, as well as which alterations may occur in the autonomic modulation of these individuals.

Data such as these are important, because, in addition to adding elements to the literature related to the abovementioned topic, it can determine whether this type of analysis can be a relevant tool to identify and help in the understanding of DM1 influence in the autonomic modulation.

The aim of the study was to compare HRV indexes obtained through symbolic analysis and SE, in young adults with DM1 and healthy subjects, associated with the analysis of the indexes obtained in the time and frequency domain, as well as to verify whether there are correlations between them for the young adults with DM1. The initial hypothesis was that the symbolic analysis and SE could identify autonomic alterations in individuals with DM1, when compared to healthy subjects, as well as the traditional HRV indexes, and that there are good correlations between such indexes.

## Methods

To develop this cross-sectional observational study, 43 young adults diagnosed with DM1 were enrolled by convenience and allocated to the DM1 group and 45 healthy young individuals without DM1 were enrolled in the Control Group. To participate in the study, the DM1 Group volunteers should have a clinical diagnosis of DM1, confirmed by medical diagnosis and/or blood test, and no diagnosis or clinical signs of autonomic cardiac neuropathy. Neither group should have individuals with cardiorespiratory diseases and none of them were smokers and/or alcoholics. Only series of R-R intervals with more than 95% of sinus beats were included in the study, that is, series with variations in the measurement greater than 5% were disregarded.^12^

To recruit the DM1 Group participants, endocrinologists and Basic Health Units were contacted and, for the Control group, students from a public university were invited to participate. To perform the sample calculation, considering the absence of studies with SE or symbolic analysis with DM1, the index corresponding to the square root of the mean of the squared differences between adjacent normal RR intervals (RMSSD) was used, which is a classic index in HRV analysis. For the RMSSD index, considering a difference of 19.85 milliseconds, for a standard deviation of 25.30 milliseconds,^13^ with an alpha risk of 5% and beta of 80%, the sample size resulted in at least 25 individuals for each group; however, for eventual device reading errors, this number was increased in both groups.

All procedures used in this study followed the Helsinki Declaration and were approved by the Research Ethics Committee of *Faculdade de Ciências e Tecnologia* (FCT/UNESP), Presidente Prudente *Campus* (CAAE: 22530813.9.0000.5402, opinion 417.031). The individuals signed the Free and Informed Consent Form.

Initially, the volunteers underwent an interview to collect the following information: age, gender, time of diagnosis (for diabetics) and use of medications. Body mass (Welmy R/I 200 digital scale, Brazil) and height (Sanny stadiometer, Brazil) were also measured to obtain the Body Mass Index (BMI).^14^ After these assessments, volunteers were submitted to the experimental protocol, which consisted in the assessment of autonomic modulation through heart rate (HR) monitoring, using a Polar S810i cardio-frequency meter (Polar Electro, Finland), and the use of the R-R interval series obtained for the analysis of HRV.^15^

In order to perform this stage, the volunteers were instructed to remain silent, awake, at rest, with spontaneous breathing for 30 minutes, in dorsal decubitus for HR measurement. All volunteers were instructed not to consume ANS stimulants, such as coffee, tea, soda, and chocolate, and not to perform physical activities 24 hours prior to the evaluation, in order not to interfere with cardiac autonomic modulation.

The evaluations were performed individually in a room with temperature between 21°C and 23°C, and humidity between 40% and 60%, in the afternoon, between 1 pm and 6 pm to minimize circadian rhythm influences.^16^

After HR measurement using a cardio-frequency meter, a thousand consecutive R-R intervals were selected from the period of greatest signal stability, and digital filtering was performed with a filter moderated by the software Polar Precision Performance™ SW (version 4.01.029), followed by the manual one, to eliminate premature ectopic beats and artifacts.

To evaluate the non-linear behavior of HRV, symbolic analysis and SE were used. The symbolic analysis evaluation is based on the quantification of the information carried in a temporal series, on the transformation of the previously selected R-R intervals into integers from zero to six, from which the symbolic patterns (three-symbol sequence) are constructed.

All possible patterns were grouped without loss into four families, according to the number and type of variations between subsequent symbols,^9^ namely: (1) patterns, without variation (0V), that is, three equal symbols, for instance, "4,4,4"; (2) patterns with one variation (1V), that is, two subsequent equal symbols and a different one, for instance, "4,2,2"; (3) patterns with two similar variations (2LV), that is, the three symbols form an upward or downward slope, for instance, "1,3,4" or "5,4,2"; (4) patterns with two different variations (2ULV), that is, the three symbols form a peak or a valley, for instance, "3,5,3" or "4,1,2".

The pattern related to the sympathetic branch performance is represented by the 0V family, and the performance of the parasympathetic branch is represented by the 2LV and 2ULV patterns. The joint performance of the ANS branches is represented by the 1V family.^9^ The frequencies of occurrence of these families (0V%, 1V%, 2LV% and 2ULV%) were evaluated in this study. To calculate these indexes, we counted the number of times a pattern, which belonged to a specific family, was found, using a specific non-linear analysis software.^9^

Another variable calculated in the same software was SE, which represents the complexity of pattern distribution. SE was used to quantify the complexity/regularity of heart rate fluctuations. Based on Shannon's framework, entropy is the measure of information of a given message - a message with low entropy/information is characterized by repetition.^9^

For the analysis of the linear HRV indexes, the RMSSD indexes and the standard deviation of the mean R-R intervals (SDNN) were used in the time domain.^4^

As for the frequency domain, the low (LF: 0.04 to 015 Hz) and high frequency (HF: 0.15 to 0.40 Hz) spectral components, in milliseconds squared and normalized units (n.u.), and the ratio of these components (LF/HF), were used. The spectral analysis was calculated using the Fast Fourier Transform algorithm.^4^ The HRV analysis software (Kubios, Biosignal Analysis and Medical Image Group, Department of Physics, University of Kuopio, Finland) was used to calculate these indexes.^17^

### Data analysis

In order to characterize the assessed volunteers, the descriptive statistical method was used, and the results are shown in absolute numbers, means and standard deviation for data with normal distribution (height, RMSSD, LF n.u., HF n.u., 2LV and SE) and median and interquartile range for those with non-normal distribution (age, body mass, BMI, SDNN, LF ms^2^, Hf ms^2^, LF/HF, 0V, 1V and 2ULV).

For comparison between the groups (control and DM1), the normality of the data was initially determined using the Shapiro-Wilk test. When the normal distribution was accepted, the Student’s *t* test for independent groups was applied and, in the cases where the normal distribution was not accepted, the Mann-Whitney test was applied. The effect size of the difference between the comparisons was analyzed by Cohen’s d and values above 0.80 were adopted with high magnitude.^18^

In order to verify the association and agreement between the indexes, correlations were carried out between linear and non-linear HRV indexes and, for this purpose, Pearson’s correlation was applied to the data with normal distribution, or Spearman’s correlation, for the ones that did not accept this distribution. Correlation values of r from 0.7 to 1 were considered strong, 0.4 to 0.6 were considered moderate and values of 0.1 to 0.3 were considered weak. The Intraclass Correlation Coefficient (ICC) was calculated.

The statistical significance was set at 5% and the Confidence Interval at 95% (95% CI). Data analysis was performed using the software MiniTab version 13.20 (Minitab, Pa, United States) and Statistical Package for Social Sciences (SPSS), version 15.0 (SPSS Inc., Chicago, IL, United States).

## Results

Of the 88 assessed volunteers, six showed errors in the R-R interval series greater than 5% and were excluded. Therefore, we analyzed the data of 39 young adults with DM1 (20 females) and 43 healthy young individuals (22 females), whose characteristics can be seen in table 1. The DM1 group had higher values of body mass and BMI (p < 0.05).

**Table 1:** Characteristics of the control and type 1 diabetes mellitus (DM1) groups

Variables	Control (n = 43)	DM1 (n = 39)	p value
Age, years*	21.00 (5.00)	21.00 (7.00)	0.5397
Body mass, kg*	60.30 (22.80)	68.15 (22.90)	0.0129
Height, m†	1.69 (0.09)	1.73 (0.17)	0.4680
BMI, kg/m^2^*	22.19 (4.67)	24.19 (5.84)	0.0216

Values in bold represent p < 0.05.

* Median (interquartile range);

† mean (standard deviation). BMI: body mass index.

Of the young individuals with DM1, 38.46% used other drugs in addition to insulin. Medications for the control of blood pressure (12.82%) and cholesterol (7.69%) were also used by these individuals. Moreover, 20.51% used drugs for thyroid disorders, 12.82% used contraceptives and 20.51% used medications for several diseases, such as rhinitis, diabetic polyneuropathy, peripheral neuropathy and epilepsy.

Table 2 shows the linear index values in the HRV time domain for both groups. Significantly lower values were observed in the DM1 group when compared to the control group for both indexes (SDNN and RMSSD). The effect of the difference between the groups was considered high, as demonstrated by the obtained value of d = 1.210 and d = 1.203 for the SDNN and RMSSD indexes, respectively.

**Table 2:** Values of the heart rate variability linear indexes in the time domain of the control and type 1 diabetes mellitus (DM1) groups

Index	Control (n = 43)	DM1 (n = 39)	p value	Cohen's d Effect size	
SDNN*	64.50 (36.20)	37.30 (29.90)	0.0001	1.21	Large
RMSSD†	55.59 (21.60)	32.73 (17.43)	0.0001	1.203	Large

Values in bold represent p < 0.05.

* Median (interquartile range);

† mean (standard deviation). SDNN: standard deviation of all normal R-R intervals recorded in a time interval, expressed in milliseconds; RMSSD: the square root of the mean of the squared differences between adjacent normal R-R intervals, in a time interval, expressed in milliseconds.

Table 3 represents the linear index values in the HRV frequency domain for both groups. Significantly lower values were observed in the DM1 group when compared to the control group for the LFms^2^ and HFms^2^ indexes. Regarding the effect of the difference between the groups, we obtained d = 0.9703 for the LFms^2^ index, showing a large effect, and, for the HFms^2^ index, d = 0.7759, considered a medium effect. For the other indexes, the effect was considered small, with d < 0.2.

**Table 3:** Linear index values of heart rate variability in the frequency domain of the control and type 1 diabetes mellitus (DM1) groups

Index	Control (n = 43)	DM1 (n = 39)	p value	Cohen's d Effect size	
LF ms^2^*	1,203.00 (1148,00)	402.00 (531.00)	0.0001	0.9703	Large
HF ms^2^*	963.00 (866.00)	386.00 (583.00)	0.0001	0.7759	Medium
LF n.u.†	49.76 (16.72)	54.54 (14.83)	0.1770	-0.332	Small
HF n.u.†	50.23 (16.72)	45.45 (14.84)	0.1770	0.3325	Small
LF/HF*	0.97 (1.05)	1.13 (0.82)	0.3071	-0.537	Small

Values in bold represent p < 0.05.

* Median (interquartile range);

† mean (standard deviation). LF: low-frequency component; HF: high-frequency component.

Table 4 shows the values obtained with the HRV symbolic analysis and SE for both groups. Significantly lower values were observed in the DM1 group when compared to the Control group for the 2ULV index, but this difference was considered as having a small effect (d = 0.4803).

**Table 4:** Values of the symbolic analysis and Shannon entropy of the control and type 1 diabetes mellitus (DM1) groups

Index	Control (n = 43)	DM1 (n = 39)	p value	Cohen's d Effect size	
0V%*	17.93 (13.33)	23.04 (18.24)	0.1290	-0.352	Small
1V%*	47.69 (7.62)	48.79 (4.81)	0.4920	-0.091	Small
2LV%†	11.99 (6.49)	11.73 (6.71)	0.8613	0.0393	Small
2ULV%*	20.24 (12.73)	15.33 (9.22)	0.0114	0.4803	Small
ES†	3.74 (0.40)	3.61 (0.45)	0.1663	0.3053	Small

Values in bold represent p < 0.05

* mean (standard deviation);

† median (interquartile range). 0V: pattern without variation; 1V: pattern with 1 variation; 2LV: pattern with two similar variations; 2ULV: pattern with two different variations; SE: Shannon entropy.

Table 5 shows the values of r, ICC and 95%CI obtained at the correlation of the linear indexes with SE and the indexes obtained in the symbolic analysis for the DM1 Group. The agreement shown by the ICC values was considered moderate for the associations of the RMSSD, SDNN, HF n.u. indexes with the 2LV index. The same was found regarding the association of RMSSD and HF n.u. indexes with the 2ULV index. High agreement was found for the LF n.u. index with the 0V index. Additionally, the 0V, 2LV, 2ULV and SE indexes showed a moderate correlation with SDNN, RMSSD, LF and HF indexes in ms^2^ and n.u. and the LF/HF ratio, with the exception of the 2ULV index with the SDNN index, SE with the HFms^2^ index, and 0V with the HFms^2^ index, which showed a weak correlation. A strong correlation was found for the 0V indexes with LF n.u., HF n.u. and LF/HF, for the 2LV index with LFms^2^ and for the SE index with LF/HF. The 1V index was the only one that did not show a significant correlation with the other HRV linear indexes and the 2ULV only for the HFms^2^ index.

**Table 5:** Association of linear and nonlinear indexes of heart rate variability in the group with type 1 diabetes mellitus

Index	0V%	1V%	2LV%	2ULV%	SE
**SDNN**					
r	-0.494	0.043	0.522	0.357	0.478
ICC	-1.204	0.058	0.410	0.295	0.037
95%CI	-3.204- -0.156	-0.795-0.506)	-0.126-0.690	-0.345-0.630	-0.836-0.495
**RMSSD**					
r	-0.690	-0.081	0.550	0.627	0.650
ICC	-3.337	-0.072	0.539	0.617	0.065
95%CI	-7.271- -1.274	-1.045-0.438	0.122-0.759	0.270-0.799	-0.782-0.510
**LF ms^2^**					
r	-0.692	0.034	0.721	0.495	0.669
ICC	-0.054	0.000	0.000	0.021	0.002
95%CI	-1.009-0.447	-0.907-0.476	-0.849-0.491	-0.866-0.487	-0.903-0.477
**HF ms^2^**					
r	-0.365	0.036	0.469	0.202	0.381
ICC	-0.032	0.001	0.020	0.010	0.001
95%CI	-0.967-0.459	-0.905-0.476	-0.869-0.486	-0.888-0.481	-0.905-0.476
**LF n.u.**					
r	0.776	-0.080	-0.638	-0.601	-0.640
ICC	0.871	-0.295	-1.840	-1.575	-0.081
95%CI	0.754-0.932	-1.470-0.321	-4.416- -0.489	-3.911- -0.350	-1.062-0.433
**HF n.u.**					
r	-0.776	0.080	0.638	0.601	0.640
ICC	-6.750	0.228	0.648	0.612	0.075
95%CI	-13.779- -3.064	-0.473- 0.595	0.329-0.815	0.260-0.796	-0.764-0.515
**LF/HF**					
r	0.776	-0.080	-0.688	-0.601	-0.704
ICC	0.321	-0.712	-0.658	-0.294	-0.702
95%CI	-0.294-0.644	-2.264-0.102	-2.161-0.131	-1.468-0.321	-2.246-0.107

Values in bold represent p < 0.05. 0V: pattern without variation; 1V: pattern with a variation; 2LV: pattern with two similar variations; 2ULV: pattern with two different variations; SE: Shannon entropy; SDNN: standard deviation of all normal R-R intervals recorded in a time interval, expressed in milliseconds; ICC: intraclass correlation coefficient; 95% CI: 95% confidence interval; RMSSD: square root of the mean of the squared differences between adjacent normal R-R intervals, in a time interval, expressed in milliseconds; LF: low frequency component; HF: high frequency component; n.u.: normalized units.

## Discussion

The aim of this study was to compare HRV indexes obtained through symbolic analysis and SE in young adults with DM1 and healthy young individuals, associated with the analysis of indexes obtained in the time (RMSSD and SDNN) and frequency (LF, HF in ms^2^ and in n.u., and LF/HF) domains, as well as verifying the existence of correlations between them in diabetic individuals.

Our results show that individuals with DM1 have a reduction in the parasympathetic (RMSSD, HFms^2^ and 2ULV), sympathetic (LFms^2^) and global (SDNN) activities of the ANS. They also showed that the 0V (sympathetic), 2LV (parasympathetic), 2ULV (parasympathetic) and SE indexes have moderate correlation with the SDNN, RMSSD, LF and HF indexes in ms^2^ and n.u. and LF/HF ratio, with the agreement of this association being considered moderate for the RMSSD, HF n.u., 2LV and 2ULV indexes, and high for LF n.u. and 0V indexes.

The individuals with DM1 also had higher values of body mass and BMI in relation to the healthy subjects, which was also shown in the study of Javorka et al.,^19^ for the BMI variable, when evaluating 17 young adults (22.4 ± 1.0 years) with DM1.

Based on the analysis of the HRV, it was possible to observe significantly lower values in the DM1 Group when compared to the Control, both for the SDNN and RMSSD indexes in the time domain and for the LFms^2^ and HFms^2^ indexes in the frequency domain, indicating a reduction in the global, sympathetic and parasympathetic activity of the ANS, with the effect of this difference being considered medium (HFms^2^) and large (SDNN, RMSSD and LFms^2^).

Similar results were indicated by other studies^19-21^. Jaiswal et al.,^20^ when evaluating 354 young individuals with DM1 (18.8 ± 3.3 years), observed a reduction in the SDNN, RMSSD, HF n.u. LF n.u. indexes and LF/HF ratio. Javorka et al.,^19^ evaluated a smaller sample of 17 individuals with DM1 (22.4 ± 1.0 years), indicating that it was possible to observe a significant reduction in the SDNN, RMSSD, LFms^2^ and HFms^2^ indexes. Also, Dungan et al.,^21^ when evaluating 33 individuals with DM1 older than 18 years, also found a reduction in the HFms^2^ index. These results show there is an autonomic alteration in DM1.

Despite the similar results, our study did not show a statistically significant difference between the groups when analyzing the LF and HF indexes in normalized units and in the LF/HF ratio.

Other methods of HRV analysis addressed in this study were non-linear: SE and symbolic analysis. This was recently described by Porta et al.^9^ and is based on the quantification of the information carried in a time series, allowing the development of patterns, denominated symbols (0V, 1V, 2LV and 2ULV), through specific calculations^9^ that indicate the autonomic behavior.

The values obtained with the symbolic analysis in this study were significantly lower in the DM1 group when compared with the control for the 2ULV index, showing a parasympathetic decrease, although the magnitude of this difference was small. A parasympathetic reduction through symbolic analysis has also been observed by other authors.^10,19^

Javorka et al.,^19^ when comparing the symbolic analysis in young adults with DM1 (21.9 ± 0.9 years) with healthy subjects of the same age group, observed a reduction of 2LV%, which reflects sympathetic and parasympathetic modulation, with parasympathetic predominance. A similar result was observed in the study by Moura-Tonello et al.^10^ who, when comparing adults (50.53 ± 6.96 years) with DM2 with healthy individuals, showed that, in diabetic individuals, there was a 2LV% reduction and an increase of 0V%, which reflects the sympathetic activity.

Regarding SE, used to quantify the complexity/regularity of heart rate fluctuations,^8^ no statistically significant difference was found when comparing the DM1 group with the healthy group. Corroborating our results, other authors also pointed out the lack of alteration in the complexity evaluated by SE, both in adults with DM2^10^ and in young individuals with DM1.^19^ These results suggest that, despite the autonomic imbalance, identified through other HRV indexes, the autonomic complexity seems to have no influence in these populations.

Another point addressed in this study was the existence of an agreement in the correlations between linear and non-linear indexes (symbolic analysis). Moderate agreement was observed in the correlations of the RMSSD and HF n.u. indexes with the 2ULV and 2LV indexes of the symbolic analysis, and SDNN with 2ULV, in addition of a high concordance in the correlation between the LF n.u. and 0V indexes. These results demonstrate that the indexes obtained through the symbolic analysis show moderate and high agreement with the indexes obtained in the time and frequency domains, indicating they can also be used in the ANS analysis, since they showed similar results to those of the linear analyses and were able to identify changes in autonomic modulation, as well as the traditional HRV indexes.

HRV alterations have been pointed out as a strong indicator of risk related to cardiovascular events, both in healthy individuals and in those with an already established disease.^22^ This condition increases the risk of sudden death due to cardiac arrhythmias and is associated with elevated mortality rates from other causes,^23^ indicating that cardiac autonomic dysfunction in patients already at risk, such as those with DM, may be a complicating agent.^24^ HRV reduction is described as the first sign of CAN,^25^ with a mortality rate that is five-fold higher than in patients without this complication,^26^ being suggested as a diagnostic test by the American Diabetes Association® (ADA).^26^

Some limitations should be pointed out, such as the cross-sectional study design, which made it impossible to follow the individuals for a longer time, not allowing to know whether the influence of DM1 on the ANS would remain or worsen in the long term. Also, the anthropometric characteristics, such as weight and BMI, were different between the groups and higher in the group with DM1; however, the mean values were within the normal range, that is, below the values considered for obesity and overweight (BMI < 25 kg/m^2^).^14^

Despite the abovementioned limitations, some positive points should be emphasized, such as the use of new methods of non-linear HRV analysis, such as the symbolic analysis, which was able to identify autonomic alterations in DM1 and can be used to evaluate and monitor this population. Moreover, we observed that the symbolic analysis showed a moderate and high agreement with some indexes evaluated in this study, indicating that this method should be included in the HRV analysis, associated with the traditional indexes of the time and frequency domains, being crucial in the clinical follow-up of the ANS status in this population. However, considering the small number of studies with this population that used methods such as symbolic analysis, further research should be encouraged so that more information can be disseminated about this method in other age groups and populations.

## Conclusion

The results show that type 1 diabetes mellitus influences linear indexes (time and frequency domains) and the symbolic analysis; however, it does not yet influence the heart rate variability complexity. Symbolic analysis correlates with linear indexes of heart rate variability.
